# SEQ2MGS: an effective tool for generating realistic artificial metagenomes from the existing sequencing data

**DOI:** 10.1093/nargab/lqac050

**Published:** 2022-07-25

**Authors:** Pieter-Jan Van Camp, Aleksey Porollo

**Affiliations:** Department of Biomedical Informatics, University of Cincinnati, Cincinnati, OH, USA; Division of Biomedical Informatics, Cincinnati Children's Hospital Medical Center, Cincinnati, OH, USA; Division of Biomedical Informatics, Cincinnati Children's Hospital Medical Center, Cincinnati, OH, USA; Center for Autoimmune Genomics and Etiology, Cincinnati Children's Hospital Medical Center, Cincinnati, OH, USA; Department of Pediatrics, University of Cincinnati, Cincinnati, OH, USA

## Abstract

Assessment of bioinformatics tools for the metagenomics analysis from the whole genome sequencing data requires realistic benchmark sets. We developed an effective and simple generator of artificial metagenomes from real sequencing experiments. The tool (SEQ2MGS) analyzes the input FASTQ files, precomputes genomic content, and blends shotgun reads from different sequenced isolates, or spike isolate(s) in real metagenome, in desired proportions. SEQ2MGS eliminates the need for simulation of sequencing platform variations, reads distributions, presence of plasmids, viruses, and contamination. The tool is especially useful for a quick generation of multiple complex samples that include new or understudied organisms, even without assembled genomes. For illustration, we first demonstrated the ease of SEQ2MGS use for the simulation of altered Schaedler flora (ASF) in comparison with *de novo* metagenomics generators Grinder and CAMISIM. Next, we emulated the emergence of a pathogen in the human gut microbiome and observed that Kraken, Centrifuge, and MetaPhlAn, while correctly identified *Klebsiella pneumoniae*, produced inconsistent results for the rest of real metagenome. Finally, using the MG-RAST platform, we affirmed that SEQ2MGS properly transfers genomic information from an isolate into the simulated metagenome by the correct identification of antimicrobial resistance genes anticipated to appear compared to the original metagenome.

## INTRODUCTION

High-throughput shotgun DNA sequencing (HTS) enables the analysis of many different microbiome environments (1–5). In the context of a human body, HTS allows for determining the variability of microbiota in healthy individuals at different body sites (6, 7) and finding associations of microbiome changes with various conditions including diabetes, obesity, or even cancer (8–10). Whole genome sequencing (WGS) appears to be more advantageous than 16S rRNA amplicon sequencing in many ways (11). Beside obvious expansion to viral and fungal kingdoms, WGS allows for better delineation of microbes in a microbiome due to availability of genomic data outside of the 16S rRNA gene. This is especially useful for metagenomics studies that require gene-level resolution analysis such as early detection of an emerging pathogen and its antimicrobial resistance profile, identification of strains and sequence types and classification of their virulence, and many other applications. However, even metagenomics tools for taxonomic classification (MTTC) that use WGS suffer the loss of accuracy below the genus level, as the delineation of highly similar organisms becomes increasingly difficult (12). Furthermore, since new species or strains are constantly being sequenced, the reference databases need to be updated regularly.

When creating metagenomics analytical pipelines or tools, it is important to have annotated benchmark datasets in which the content of the metagenome is (at least partially) known with the aim of testing a hypothesis or validating the workflow. There have been some *in vivo* experiments when germ-free environments were colonized by a selected set of microorganisms in an attempt to create a pre-defined metagenome with all constituents known. An example is the altered Schaedler flora (ASF), originally created in the 1970s, where eight naturally occurring intestinal microorganisms were grown in sterilized mice's intestines (13). It provides a well characterized metagenome composition in a real host environment that is relatively stable and passed through multiple generations. The gnotobiotic models like this are important for evaluating the complex interactions between species in a microbiome and with the host (14–16). However, these types of experiments are expensive, laborious and limited in complexity. Furthermore, validating a bioinformatics pipeline requires diverse data with many permutations to assure good accuracy and generalization, making the controlled *in vivo* or *in vitro* culturing of microorganisms in different environmental conditions impractical (17).

To evade the need for acquiring microbiome environments, various bioinformatics tools were developed that generate the artificial metagenome sequencing files to emulate the output of real sequencing experiments (see the summary in (18)). These tools, which we will call *de novo* sequencing read generators (DNG) in this work, simulate sequencing reads from scratch using various statistical models to sample pieces of DNA from complete or partially assembled reference genomes. These sampled sequences may be further altered to emulate sequencing errors, mutations, variation in read length, or uneven distribution of the genome coverage to approach the real-world output of the sequencing machines. However, tuning the hyperparameters of DNG (e.g. read length, mutation rate, gene copy variation, experimental biases, etc.) to achieve a more realistic metagenome may be a very tedious task. Moreover, the need to provide reference genomes for blending makes it difficult to compose comprehensive metagenomic samples that most likely may contain plethora of viruses, unannotated organisms or strains, plasmids, and DNA contamination that constitute real experimental samples.

In this work, we present SEQ2MGS, a simple yet very effective generator of artificial metagenomes that does not depend on simulating reads *de novo* from a set of reference genomes, but rather samples the reads from real sequencing experiments. The tool either blends shotgun reads from different isolates together or mixes one or more isolates in the actual sequenced metagenome. SEQ2MGS goes beyond simple permutations of concatenating and shuffling the samples. It enables the control for genomic proportions of both individual blended isolates and metagenomic background, addressing the pursued relative abundance or genomic coverage. The easy setup of the execution is amenable to the quick automated generation of multiple metagenomics experiments.

We envision that SEQ2MGS will be useful when the complexity of artificial data and realism of composition are more important than the control for detailed content. By sampling and mixing the reads from various existing sequencing experiments, the produced artificial sample reflects all the variations and imperfections intrinsic to sample processing, sequencing equipment, potential contamination, and underlying genomic complexity. Examples of applications include the benchmarking of computational methods for limits of detection of an organism or genes of interest from a human or environmental metagenomics samples, evaluating the effects of contamination of samples on existing pipelines, or the emulation of emergence of a pathogen for training the surveillance systems based on high-throughput sequencing. In other words, this tool enables the assessment of computational analytical workflows with the focus on a few select organisms or genes that should be derived from complex, realistic environments, and fills the niche in metagenomics where *de novo* generators might fall short.

## MATERIALS AND METHODS

### SEQ2MGS workflow

**Figure 1.**
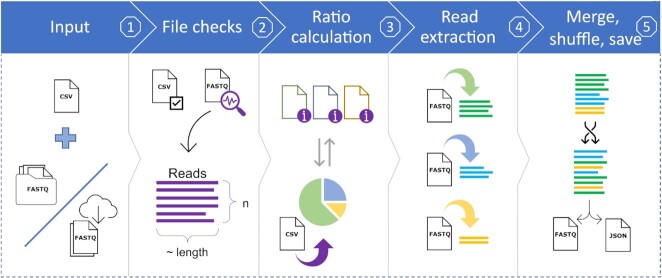
Overview of the SEQ2MGS pipeline. **Step 1**. Input is provided in the comma-separated values (CSV) formatted table that specifies details on each sequencing data file (FASTQ) to be used: source (local or web), targeted genomic proportions. **Step 2**. Input is validated, files are downloaded if requested, and sequencing reads in each file are assessed for the total number and the average read length. **Step 3**. The number of reads needed from each file is estimated by accounting for their corresponding distribution in the original files and the targeted proportion in the resulting file. **Step 4**. Reads needed from each file are randomly sampled. **Step 5**. The drawn reads are combined and shuffled to create the final FASTQ file. A JSON file with details on the parameters used and characteristics of both input and output data is generated alongside the data for referencing.

Figure 1 illustrates the SEQ2MGS workflow. SEQ2MGS operates on a set of existing FASTQ files; each may represent a sequenced isolate (i.e. an individual organism) or a metagenome. These files are accepted from both local sources and the public repository Sequencing Reads Archive (SRA) at the National Center for Biotechnology Information (NCBI), provided the SRA Toolkit (19) is installed.

SEQ2MGS takes the input parameters from a CSV file with at least four columns: (i) Absolute paths to the sequencing files or SRA accession ID. (ii) Type of the file, which is either an isolate (I) or background (B). The latter is used when an existing sequencing file (e.g. of metagenome) is used to mix in isolates of interest. (iii) Targeted relative abundance (RA) or coverage of isolates. In case of RA, if all files are marked as isolates, the sum of the RA column must be equal to 1. If a background file is set, the sum of the abundances of the isolates must be <1. When an isolate is defined by coverage, its genome coverage in the final mix must be specified, e.g. 15X. (iv) The estimated size of the isolate's genome in basepairs (bp). This is needed to assure correct calculations in case of specified coverage. Details on all inputs and optional parameters are described in the SEQ2MGS documentation.

In each specified FASTQ file, SEQ2MGS counts the number of reads and their average length. The latter is important as different samples may be sequenced with different read lengths, which needs to be accounted for, when estimating the number of reads required from each source to achieve the goal of genomic content measured in nucleotide bases.

The equations below estimate the number of reads needed for a composition depending on the pursued scenario, i.e. defined by the target genome coverage or relative abundance of organisms, also in the absence or presence of the background microbiome.

For compositions of two or more isolates defined by coverage:

(1) $$\begin{equation*}R{N_i} = \ \frac{{G{S_i} \times G{C_i}}}{{R{L_i}}},\end{equation*}$$

where *RN_i_* is the number of reads needed from the isolate $i \in I,\ I\ = \{ {1,\ 2, \ldots ,n} \}$; *GS_i_* and *GC_i_* are its genome size and genome coverage, respectively (to be specified by the user); *RL_i_* is the average read length in the corresponding FASTQ file (to be automatically found by the tool).

For the same compositions but in the presence of the microbiome background (*B*), the number of background reads (*RN_B_*) needed is:

(2) $$\begin{equation*}R{N_B} = \ \frac{{BB}}{{R{L_B}}},\end{equation*}$$

where *BB* is the number of background bases defined in Eq. (3) that accounts for replacement of microbiome reads with those from isolates to keep the original size of the genomic content; *RL_B_* is the average read length automatically computed by SEQ2MGS from the metagenome source file.

(3) $$\begin{eqnarray*}BB\ &=& 0 < minBB \le R{C_B}\ \times \ R{L_B}\nonumber\\ && - \mathop \sum \limits_{i\ \in \ I} R{N_i} \times R{L_i} \le maxBB,\end{eqnarray*}$$

where *minBB* and *maxBB* are the optional lower and upper limits of *BB*, respectively, not used if not specified; *RC_B_* is the total read count in the metagenome source file (automatically computed by the tool).

For compositions of two or more isolates defined by genomic relative abundance (*RA*), in the absence or presence of the background metagenome, the required numbers of reads for each component are defined as follows:

(4) $$\begin{equation*}R{N_k} = \ \frac{{TB\left( x \right) \times R{A_k}}}{{R{L_k}}}\end{equation*}$$

(5) $$\begin{equation*}TB\left( x \right)\ = \Bigg\{ \begin{array}{@{}*{1}{c}@{}} {0 < minTB \le \mathop {\min }\limits_{i\ \in \ I} \left( {\frac{{R{L_i} \times R{C_i}}}{{R{A_i}}}} \right) \le maxTB,\ if\ x = 0}\\ {0 < minTB \le R{C_B} \times R{L_B} \le maxTB,\ if\ x = 1} \end{array}\ ,\end{equation*}$$

where $k \in \{ {I,\ B} \}$; *TB(x)* is the total number of bases in the target genomic content, it depends on the presence (*x* = 1) or absence (*x* = 0) of background in the designed composition. *minTB* and *maxTB* are the optional lower and upper limits of the target genomic content, respectively, not used if not specified.

Once the number of reads needed from each file is known, they are randomly sampled from the original files. If the number of reads needed from a file exceeds the number of actually available, reads are randomly resampled until the target number is reached. When resampled, the read's ID will be changed to ensure uniqueness, but the sequence will remain unchanged. Once all reads are sampled from different files, they are shuffled and concatenated into the final FASTQ file. The sampling, renaming, and shuffling of reads are performed using the BBMap package, part of the BBTools tool suite from the Department of Energy Joint Genome Institute (https://jgi.doe.gov/data-and-tools/bbtools/). The resulting sequence file is accompanied with a JSON metadata file that contains all settings and file properties (reads used, total bases, percentage of a file used, etc.). SEQ2MGS also keeps an SQLite backend database that holds the same information as in the individual JSON files but with additional logs of use of the tool, encountered errors and the read counts and lengths from all FASTQ files used. The latter information saves execution time, if the same sequencing files are used as input in multiple mixing experiments, since SEQ2MGS does not need to re-count the number of available reads and their average length.

### Tools for simulation of metagenomics data

Since there are no other established tools that sample reads from existing files, two DNG tools were chosen to compare with SEQ2MGS. Grinder can generate artificial metagenomes from a profile of provided genomes and their abundances (20). It requires a set of genomes in the FASTA format and a simple table listing the desired relative abundances for each genome. Although multiple genomes can be used as input, they have to be provided in one FASTA file and, if the assembly is fragmented (e.g. represented by multiple contigs), the relative abundances have to be split according to the contig size to ensure proper distribution in the final file. The number of total reads and their length are specified as parameters. Grinder provides simulation of several sequencing error profiles. In this work, the Illumina platform error function was chosen for consistency with other tools and also because it is suggested in the documentation. For quality scores, the suggested values of 30 and 10 were chosen to represent a good and a bad score, respectively. The complete input settings can be found in Supplementary materials S1.

CAMISIM was developed as part of the Critical Assessment of Metagenome Interpretation (CAMI) challenge and is one of the latest and most versatile and fast tools available (18, 21). It also uses genomes in the FASTA format as input. In this study, the Illumina platform was chosen for simulation. Settings for read lengths and error profiles were set to defaults. The detailed configuration settings can be found in Supplementary materials S1.

### Tools for taxonomic analysis

Metagenomics tools for taxonomic classification (MTTC) tackle the problem of detecting the organisms present in sequenced microbiome samples and/or estimate taxon abundance by mapping the reads or derived k-mers to a reference database. Some tools consider whole genomes of species as a reference, while others focus on coding regions only, or use custom genomic markers that would uniquely identify a species (22–24). There have been several studies published to benchmark performance of the most commonly used MTTC, in terms of both accuracy and resource usage (e.g. time and memory requirements). Taxonomic classification of metagenomes is still an active area of research. There is no one tool that clearly stands out, but there are differences in sensitivity, false positive rates, or resource requirements that may warrant the use of different tools in different situations (12, 25).

To compare the generated metagenomics files, three highly cited MTTC were employed: Centrifuge (24), Kraken (version 2) (23) and MetaPhlAn (version 3) (22). These three tools represent alternative approaches to the metataxonomic classification and employ different reference databases. Centrifuge was set up with its standard database of human genome, prokaryotic and viral genomes, Kraken—with the full bacterial database, and MetaPhlAn—with its default database as of 10 April 2021 (latest at the time). All tools were run with their default settings, with Centrifuge having an additional step to convert its report into the Kraken style report. Note, the aim of this comparison is not the validation of the MTTC, but rather evaluating the concordance in simulated metagenomes between DNG and SEQ2MGS and their impact on MTTC results.

The analysis of the MTTC reports was carried out at the species level using the taxonomic ID for comparison between files. Reads were counted by clade to ensure any descendants would be incorporated. The number of clade reads per detected species was converted to a percentage to be able to compare between files with different depths and used to calculate the normalized Canberra distance (26) (NCD, Eq. 6) between the generated samples as a measure of (dis)similarity.

(6) $$\begin{equation*}NC{D_{UV}} = \frac{1}{S}\ \mathop \sum \limits_{j\ = \ 1}^S \frac{{\left| {{X_{Uj}} - \ {X_{Vj}}} \right|}}{{{X_{Uj}} + \ {X_{Vj}}}},\end{equation*}$$

where *S* is the number of detected taxons, *U* and *V* are the samples to compare, and *X* is the abundance per taxon in the samples.

Visualization of the taxonomic distributions as Sankey diagrams was performed using Pavian (27) with default settings.

### Data availability/novel programs, software, algorithms

The SEQ2MGS tool is publicly available on GitHub with detailed instructions on setup and use: https://github.com/pieterjanvc/seq2mgs.

## RESULTS

We illustrate different applications of the developed SEQ2MGS pipeline by performing three experiments that compare performance of SEQ2MGS to the existing DNG tools and highlight its novel and unique features. The first experiment demonstrates the ability to mix a set of known isolates of commensal bacteria from different sequencing experiments together to generate a fully annotated intestinal metagenome. The second experiment spikes an annotated isolate of a bacterial pathogen into a separately sequenced unannotated metagenome. The third experiment shows that SEQ2MGS carries over specific genetic features annotated in the original bacterial isolate into the artificial metagenome.

### Experiment 1. simulation of altered schaedler flora

**Figure 2.**
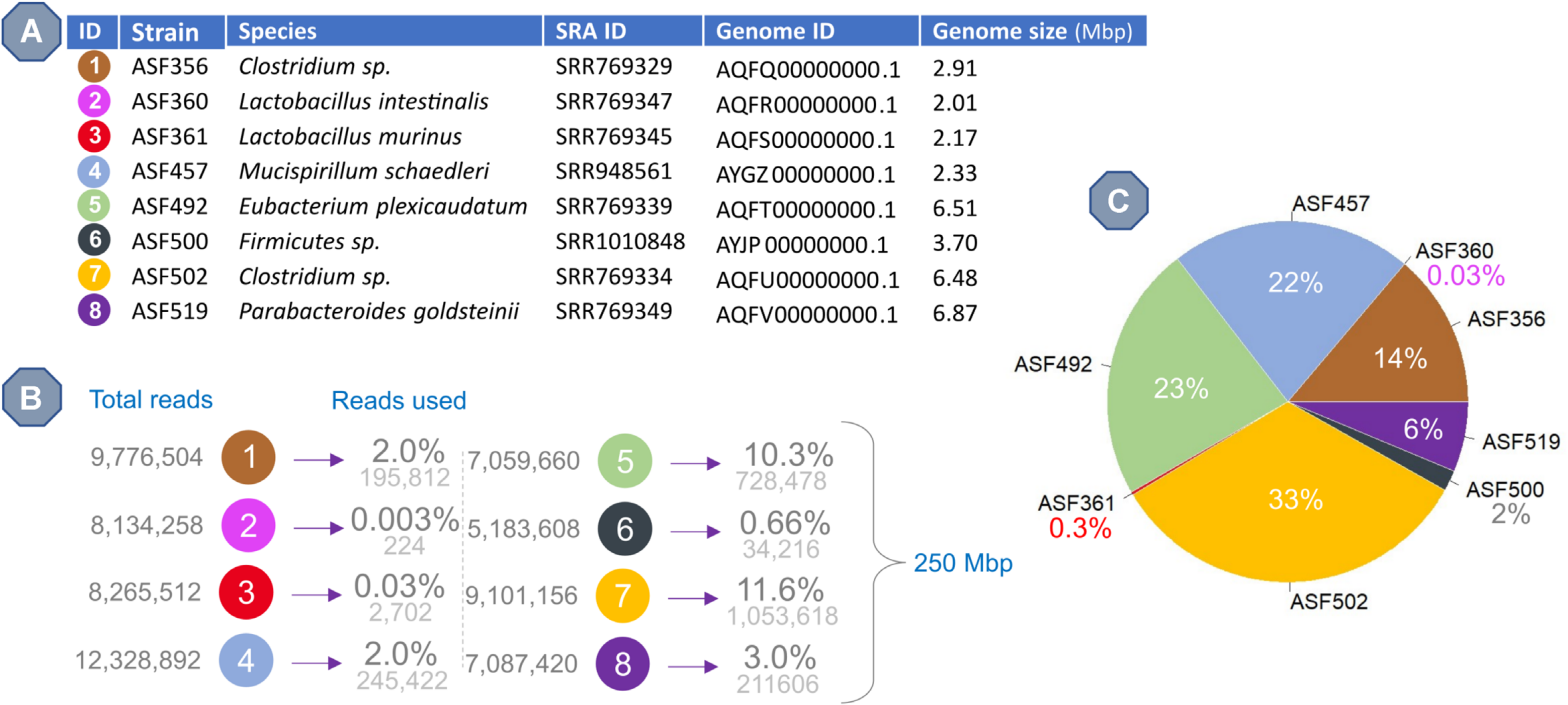
Design of Experiment 1. (**A**) Metadata on the files used to recreate the Altered Schaedler Flora. SRA ID and Genome ID columns refer to the NCBI sequencing reads file and genome accession IDs, respectively. (**B**) Summary of the calculations performed by SEQ2MGS to generate a file (250 Mbp) with the targeted relative abundances. (**C**) Graphical representation of the relative abundance of each strain as defined in the L1-M3 model by Sarma-Rupavtarm et al. (28), but adjusted for adding a trace amount of ASF360 (not detected in the original experiment).

ASF is a mouse intestinal microbiome model that consists of 8 known bacterial isolates that have been transplanted into murine sterilized guts. Sarma-Rupavtarm *et al.* performed the *in vivo* evaluation of the relative abundances of ASF in various intestinal locations (28). We defined the RA for our experiments by converting the ratios of different species from their L1-M3 model (L1 denotes the location in colon where the sample was taken, M3 is the mouse used), which was randomly chosen from the nine published in (28). Of note, the ASF360 *Lactobacillus intestinalis* strain was not detected in their experiment having been suggested to be of a too low abundance. To be consistent with the initial settings of the experiment, we still mixed in a very low amount of this species by using a tenth of the lowest abundant species (ASF361). Figure 2 summarizes the design of Experiment 1.

The sequencing data needed to recreate the ASF with SEQ2MGS were obtained from SRA, while the reference genomes for Grinder and CAMISIM were downloaded from the NCBI Assembly database (see Figure 2A for accession numbers). As Grinder is an older tool with relatively slow read generation compared to CAMISIM, we limited the Grinder output to 25 Mb. Both CAMISIM and SEQ2MGS can handle larger file generation thus 250 Mb files were generated, but an additional 25 Mb file was created by each, as well, to provide fair comparison to Grinder. Figure 2B depicts a schematic overview of the 250 Mb file generation using SEQ2MGS. Figure 2C shows the resulting relative abundance of all ASF species in the final metagenome.

**Figure 3.**
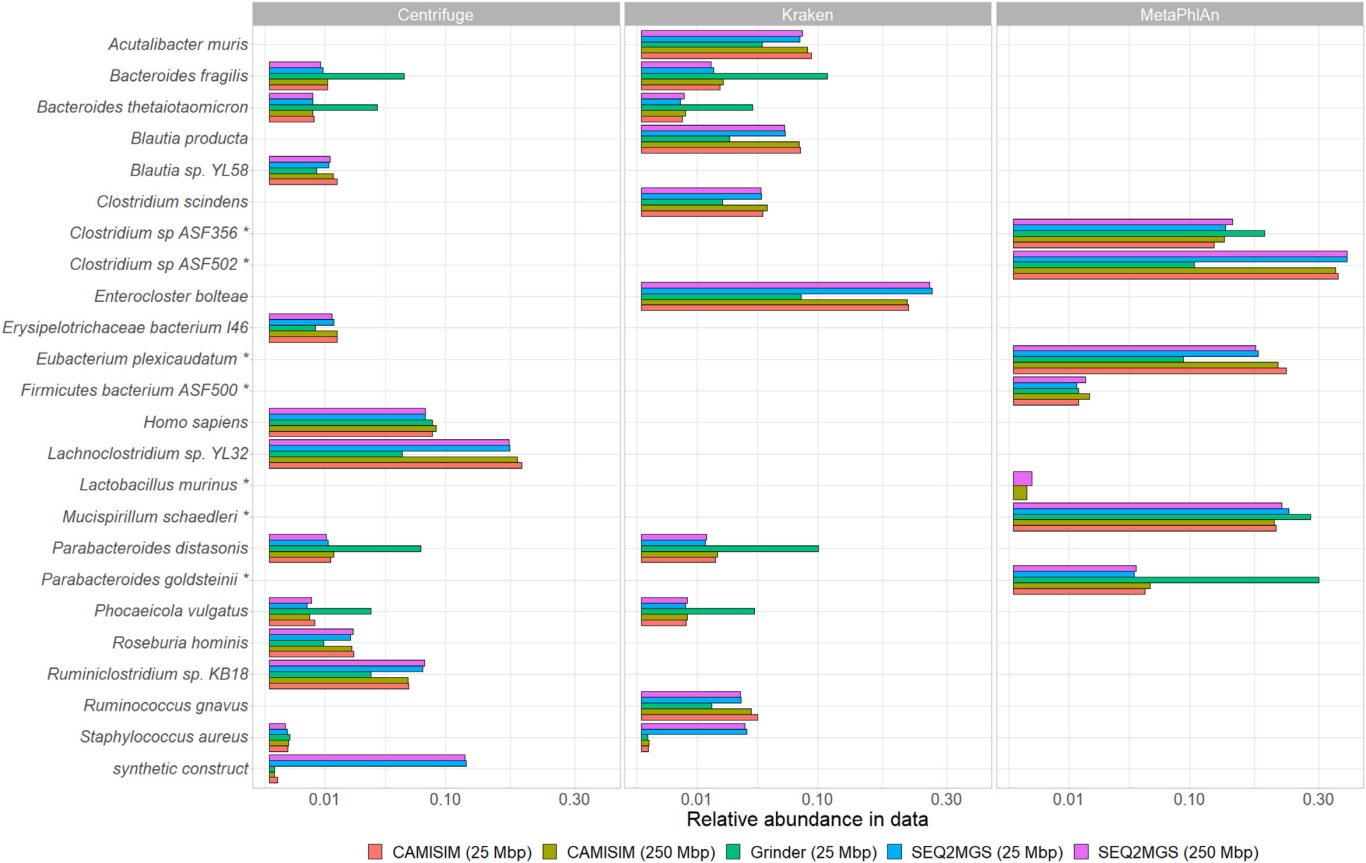
Taxonomic classification of the generated metagenome files for Experiment 1. The bar plots represent relative abundance of the top species identified by Centrifuge, Kraken, and MetaPhlAn. Color corresponds to different input files generated by CAMISIM, Grinder and SEQ2MGS. Asterisks indicate the actual ASF species and strains used for mixing. Of note, the Centrifuge database also contains human genome and markers for synthetic constructs, hence the extended report. Complete reports of the benchmarked MTTC can be found in Supplementary materials S2.

With a total of 8 species present in ASF known by design, regardless of the tool employed to generate the metagenome, both Kraken and Centrifuge vastly overestimate the number of potential species (false positives) reporting hundreds of potential hits (range 721–2564). MetaPhlAn is much more specific and only reports a handful of species per file (range 6–7). Figure 3 summarizes the reports generated by Kraken, Centrifuge, and MetaPhlAn for both DNG methods and the corresponding SEQ2MGS files. The complete output files can be found in Supplementary materials S2.

We assessed consistency between metagenome generators based on the pairwise overlap of the reported species and the normalized Canberra distance (NCD; measure of beta diversity). For the top 10 detected species, all MTTC report highly consistent results between CAMISIM and SEQ2MGS metagenomes yielding overlaps at 80%, 90% and 100% for Kraken, Centrifuge, and MetaPhlAn, respectively. Results for Grinder *vs* SEQ2MGS metagenomes considerably diverge for Kraken and Centrifuge overlapping only at 30% and 40%, respectively, though MetaPhlAn remains fully consistent. Interestingly, the overlap of top 10 species between the two SEQ2MGS files (250 and 25 Mb) is identical (100%) for all three tools. With respect to the detection of the actual species in ASF, MetaPhlAn clearly provides the most accurate prediction with the fewest false positives across all metagenome generators used.

The overlap quantification of full reports between generated ASF metagenomes is presented in Table 1. Kraken shows the largest beta diversity between the simulated samples (NCD range 0.45–0.72, whereas consistent reports should yield NCD close to 0). MetaPhlAn has the lowest NCD range 0.09–0.33. Note that the statistics for SEQ2MGS versus CAMISIM (250 Mbp files) are more favorable than comparisons to Grinder, though this might partially be influenced by the lower number of reads used in the comparison (25 Mbp files). This is further supported by looking at the comparison between the 25 and 250 Mbp files generated by CAMISIM and SEQ2MGS. The tool an input data is identical, only the file size is different, and yet the overlap in species detected by the MTTC differs significantly.

**Table 1.** Pairwise comparison of consistency of full reports by each MTTC for the simulated ASF metagenomes^a^

**Comparison of metagenome generators**	**Kraken**	**Centrifuge**	**MetaPhlAn**
SEQ2MGS versus CAMISIM	74%, 0.45	90%, 0.31	100%, 0.09
SEQ2MGS versus Grinder	42%, 0.72	62%, 0.57	100%, 0.32
CAMISIM versus Grinder	40%, 0.71	58%, 0.59	100%, 0.33
**Comparison by metagenome sizes**			
SEQ2MGS: 250 versus 25 Mb	49%, 0.65	69%, 0.47	85%, 0.17
CAMISIM: 250 versus 25 Mb	42%, 0.71	61%, 0.55	86%, 0.18

^a^The values represent percentage of species overlap followed by the normalized Canberra distance (Eq. 6).

### Experiment 2. Simulation of a gastrointestinal infection

To demonstrate one of the unique features of SEQ2MGS, compared to DNG, we simulated a scenario of a gastrointestinal infection when the gut microbiome becomes populated with an intestinal pathogen, *Klebsiella pneumoniae*. *K. pneumoniae* is a known cause of gastroenteritis and its abundance has been associated with increased risk of developing bloodstream infections (29). Early detection and quick characterization of such pathogens is one way in which metagenomics might be able to contribute to clinical practice. However, for an accurate analytical workflow like this to be created, large benchmarking datasets are needed, which are difficult to obtain due to the large number of patients required for sampling along with the laborious characterization of specific isolates of casual agents (i.e. *K. pneumoniae*). SEQ2MGS enables fast generation of metagenomes, all from a real environment, but with controlled relative abundance of one or more species mixed in, without having to go through the hurdles of generating the background itself. This type of artificial metagenomes would be very hard and time intensive to recreate with the existing DNG tools.

Here, to represent a gut microbiome background, an adult healthy metagenome stool sample was taken from a Cornell University experiment (SRA accession ID: SRR12344432). To represent the pathogen, the *K. pneumoniae* isolate sample was taken from the CDC Division of Healthcare Quality Promotion's BioProject for whole genome sequence data of Gram-negative bacteria (SRA accession ID: SRR13130411). The relative abundance for *K. pneumoniae* was set at 5%. The actual RA level at which a pathogen can become clinically relevant varies (29), but we selected a value on the low end of the spectrum to ensure plenty of other species would be in the background. This should create a more challenging benchmark set for the assessed MTTC pipelines. Thus, the resulting metagenomics file contains 30,396,456 reads, of which 980,684 reads belong to *K. pneumoniae* (3.2%). The percentage of reads is lower than the expected 5% RA because the average read length of the original *K. pneumoniae* file (227 bp) is larger than of the background file (144 bp), and therefore SEQ2MGS makes correction (3.2% × 227/144 = 5%).

**Figure 4.**
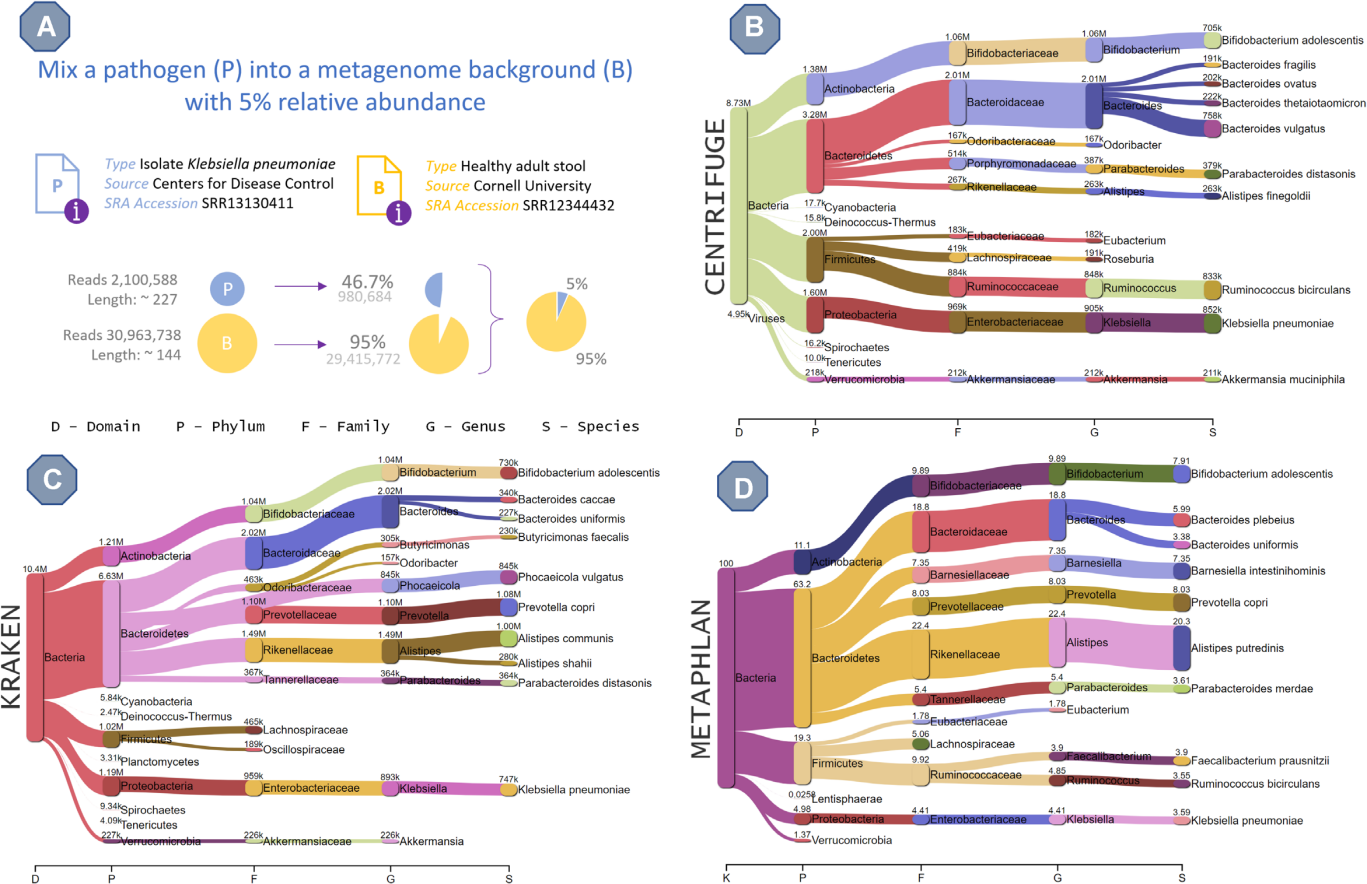
Results from Experiment 2. (**A**) Overview of the data used by SEQ2MGS to emulate an emerging intestinal infection by mixing the reads of the *K. pneumoniae* isolate into a background metagenome represented by a stool sample of a healthy individual. Note that the calculations are automatically adjusted for the difference in read length. (**B–D**) Sankey diagrams of the metataxonomic classification results by Centrifuge, Kraken and MetaPhlAn, respectively. The values at the nodes are the numbers of clade reads assigned. *K. pneumoniae* was successfully detected by all three MTTC. MetaPhlAn has the closest abundance estimate to the designed.

The generated FASTQ file was subsequently analyzed by Kraken, Centrifuge and MetaPhlAn to assess as to whether the pathogen can be properly identified and quantified from a real microbiome data. The taxonomic analyses and corresponding Sankey diagrams for the top 10 most abundant species are presented in Figure 4. All methods successfully identify *K. pneumoniae*, with 8.3%, 10.7%, and 4.4% of the clade reads detected at the species level belong to *K. pneumoniae* as estimated by Kraken, Centrifuge and MetaPhlAn, respectively.

Although the input file was identical for all assessed MTTC, comparisons at the genus and species level show dramatic differences in results between Kraken, Centrifuge, and MetaPhlAn (Table 2). For the top 10 species/genera, the overlap is 30–70% by name with NCD range 0.56–0.88. For all reported species or genera, NCD is around 1 indicating that by and large there is very low to no overlap.

**Table 2.** Pairwise comparison of MTTC reports for the human gut microbiome mixed in with *K. pneumoniae*^a^

Comparison	Top 10 species	Top 10 genera	All species	All genera
Kraken versus centrifuge	40%, 0.77	70%, 0.56	20%, 0.91	36%, 0.83
Kraken versus MetaPhlAn	40%, 0.80	60%, 0.64	1%, 0.99	3%, 0.99
Centrifuge versus MetaPhlAn	30%, 0.88	70%, 0.62	1%, 1.00	2%, 0.99

^a^The values represent percentage of species/genera overlap followed by the normalized Canberra distance (Eq. 6).

Despite the discrepancy in the overall metagenome analysis among the MTTC, the strategy of using a real metagenome sample as a background for a clinically relevant pathogen sample appears plausible for future analyses and early identification of emerging pathogens from the metagenome samples without the need for pathogen isolation via cultivation.

### Experiment 3. Metagenomics analysis beyond taxonomic classification

As metagenomics data produced by SEQ2MGS can be subjected to the same type of analyses performed on real WGS metagenomic experiments, the files can be used for more than just taxonomic classification. To demonstrate this, the data generated in Experiment 2 (*K. pneumoniae* spiked into a healthy intestinal microbiome background at 5% RA) were submitted to the MG-RAST pipeline (30). This pipeline provides both taxonomic and functional annotation of metagenomics data. Given in-depth evaluation of the metagenomic content is outside the scope of this work, we limited analysis to validating the presence of specific antimicrobial resistance genes (ARG) present in the spiked-in *K. pneumoniae* as reported by the NCBI pathogen detection project (31) using the pathogen's SRA accession ID: SRR13130411. For comparison, the original healthy background metagenome was subjected to the MG-RAST pipeline as well (i.e. not spiked with *K. pneumoniae* by SEQ2MGS).

According to the annotation by the NCBI pathogen detection project, the selected *K. pneumoniae* strain contains the following four ARG: blaSHV-60 (broad-spectrum beta-lactamase), fosA (fosfomycin resistance), emrD and oqxB19 (efflux pumps). All four ARG were detected by the MG-RAST pipeline in the spiked-in sample generated by SEQ2MGS (i.e. *K. pneumoniae* in healthy background). Per comparison, the MG-RAST results on the original healthy background metagenome did not contain these ARG apart from emrD. These results confirm that SEQ2MGS correctly transfers specific genomic information from the source files into the artificially generated data during mixing. The full pipeline reports for both files are publicly available through the MG-RAST website at https://www.mg-rast.org/mgmain.html?mgpage=project&project=mgp102827.

## DISCUSSION

This work demonstrates that SEQ2MGS can be used as an alternative to *de novo* sequencing reads generation towards achieving more comprehensive artificial metagenomes by sampling the reads from different existing sequencing experiments. The options to either mix multiple isolates or use an existing metagenome as a background, together with the flexibility of either using relative abundance or genome coverage, enable the fast and flexible generation of artificial metagenomes. SEQ2MGS also features an automated use of the vast repository of sequencing files publicly available at the NCBI Sequence Read Archive, where the mandated deposition metadata (e.g. species, anatomic site of sampling, sequencing platform, *etc*.) may facilitate the matching of samples for blending.

The strictly controlled generation of artificial metagenomes, such as the reproduction of ASF (Experiment 1), suggests that SEQ2MGS generates files comparable to DNG (CAMISIM and Grinder). Figure 3 shows that each assessed MTTC (Kraken, Centrifuge and MetaPhlAn) is consistent in its top detected species across the all input files, although Grinder is the only tool that yields RA inconsistent to other input files for a given species. It cannot be explained simply by the lower genomic content, as the 25 Mbp files generated by CAMISIM and SEQ2MGS, for comparison, do line up with their 250 Mb counterparts. On the other hand, when all detected species are compared, reports based on SEQ2MGS and CAMISIM share 74–100% of species, whereas SEQ2MGS *versus* Grinder reports are less consistent, ranging 42–100%, depending on MTTC (Table 1). Table 2, which compares the taxonomic labelling in Experiment 2, shows similar results with low overlap between the MTTC, even at the genus level. Note that in Experiment 2, all three MTTC analyzed the same input file.

We would like to emphasize, however, that the goal of this work is not to assess as to which MTTC performs better but to show the consistency of our artificially generated data with the existing established DNG. Nevertheless, results of Experiments 1 and 2 raise the question on the need for more in-depth testing of MTTC, as they seem to yield over-optimistic results in both their original publications and benchmarking studies, such as (12), where only *in silico* generated metagenomics datasets were used.

The results of Experiment 1 also demonstrate the advantage of using real sequencing data (as it is done by SEQ2MGS) for the simulation of more realistic artificial metagenomes. Specifically, by blending the sequenced ASF isolates, SEQ2MGS expectedly carries over possible sequencing contamination, such as host genome, or synthetic constructs from the library preparation step, or even DNA of a lab personnel (32–34). In fact, the latter two were detected by Centrifuge (Figure 3). However, the reads matching human may be attributed to the fact that the Centrifuge reference database does contain the mouse genome (ASF host) and, therefore, assigned those reads to the phylogenetically closest available species (human). The other two MTTC do not have any animal or human genomes in their database and thus ignored those reads. Interestingly, both CAMISIM- and Grinder-derived data also yielded human genome detected in the corresponding Centrifuge reports. This is unexpected as neither human nor mouse genomes were part of input to the DNG tools. Possible distant homology by some bacterial genomic material cannot satisfactorily explain human genome being ranked at the top 10 species. The more likely explanation is that the bacterial genomes fed to DNG have the so called *in silico* contamination with human DNA. This issue is known to cause problems but often overlooked (35).

Experiment 2 simulates an emerging pathogen within an intestinal metagenome, where we demonstrate the advantage of using our approach over DNG. To do this with a DNG approach, one first needs to elaborate an extensive list of all the species or strains present in a specific microbiome environment, which is often not fully attainable, especially given the wide variety in healthy microbiome composition. Then, one has to download all necessary genomes, while not all may be completely assembled or available altogether, along with any mobile genomic elements, such as plasmids. Next, one needs to contemplate the relative abundance of each genome to formulate the metagenome composition. Finally, multiple hyperparameters of a DNG tool have to be tuned to generate realistic reads. In SEQ2MGS, on the other hand, the setup is simply to locate an existing metagenome as a background, either as a local file or as an SRA entry, and then to mix in any additional sequenced species of interest with desired coverage or abundance. In Experiment 2, the diverse microenvironment was created with only two files in just several minutes. The input included two SRA accession IDs and the relative abundance of the pathogen in the final file. The results demonstrated that such approach can effectively generate large scale benchmark data to quickly test different analytical workflows on the identification of a targeted pathogen within a microbiome background.

Going beyond the taxonomic classification, Experiment 3 demonstrates that the data generated by SEQ2MGS properly transfers representative genomic information from the source files into the mix. This is evidenced by the MG-RAST pipeline that reports the appearance of such ARG as blaSHV-60, fosA, and oqxB19 after mixing a *K. pneumoniae* isolate into the intestinal metagenome. These three ARG, along with emrD, are attributed to the selected isolate of the pathogen. The presence of emrD in the original metagenome suggests that some other (unannotated) bacteria in the sample are likely to harbor this ARG. This is not surprising as many commensal bacteria are found to contain efflux pumps from the major facilitator superfamily (e.g. emrD) as part of their natural defense against toxic xenobiotics (36).

When working with artificial data, it is important to note that mixing reads into a sequenced background metagenome may not fully reflect the actual events occurring *in vivo*, as in the clinical course of infection, the emerging pathogen eventually alters the composition of the natural microbiome (37). This can be further confounded when a patient starts undergoing antibiotic treatment. The same challenges, though, concern the DNG-based simulations. However, with SEQ2MGS, it is much easier to emulate such background changes in composition and relative abundance, for example by borrowing the stool sequencing data of a patient undergoing antibiotic treatment for a non-intestinal infection and mixing in the isolate of a pathogen of interest with controlled amounts. It is yet another example of how SEQ2MGS can be used with sequencing experiments of people with different constitutions or diseases by taking a base level metagenome (i.e. background) and altering parts of it. This enables the tuning of analytical workflows and could serve as more realistic benchmarking.

SEQ2MGS works with any FASTQ data and thus technically can deal with the RNA sequencing data, as well. However, as gene expression levels are highly dependent on the environment (e.g. regulated by the host-microbe, microbe-microbe interactions, or other stimuli), such changes are unlikely to be simulated via a direct mix of expressed transcripts from the isolates. Simple mixtures of commensals may be still useful for benchmarking or testing, but such evaluation is beyond the scope of this work and could be addressed in future research.

It should be noted that, while FASTQ files from any origin may be used, it is advisable to combine those with comparable characteristics to prevent introducing biases: sequencing platform or library preparation protocols. This is usually provided as metadata in public repositories along with the actual sequencing files. In essence, the trade-off for the simplicity of SEQ2MGS compared to *de novo* generators is that the quality of the data chosen by the user is fully informing the quality of the resulting simulated metagenome. The user can perform quality control on the data used and process the reads using any of the wide array of tools already available. We purposefully did not include this in the workflow as research groups have different preferences in pre-processing raw data before the analysis.

While the DNG provide full control over the content of the generated reads, SEQ2MGS challenges the metagenomics analytical workflows with the same level of quality evaluation and post-processing that comes with real experimental data. Therefore, we do not consider SEQ2MGS a direct competitor to DNG tools. It rather fills the niche where the latter might fall short. Moreover, we envision that SEQ2MGS may be combined with DNG for the even more detailed generation of artificial metagenomes. For example, it is possible to mix an isolate simulated by CAMISIM into a real metagenome using SEQ2MGS. Then, the exact genome composition of the select species will be known while having a complex metagenomics background. Moreover, the CAMISIM simulation of a given isolate will enable its matching to the sequencing parameters of the background metagenome sample in case a strict match is needed but a real sample of an isolate is not available.

In conclusion, SEQ2MGS is a tool for fast and easy artificial metagenome generation by sampling reads from existing sequencing experiments and mixing them together with the targeted relative abundance or species coverage. Thus generated metagenomes may better reflect the complexities (e.g. mobile DNA elements, sequencing errors, and impurities) of real samples compared to the tools that generate reads *de novo* from reference genomes. SEQ2MGS is especially useful for quick generation of multiple samples of diverse microbiota, with possibility to include new or understudied organisms with not fully assembled or even non-available genomes, which can be used for benchmarking or early hypothesis testing when *in vivo* or *in vitro* experiments are impractical.

## DATA AVAILABILITY

SEQ2MGS is available from the GitHub repository: https://github.com/pieterjanvc/seq2mgs.

All data used during this study are available at NCBI (https://www.ncbi.nlm.nih.gov/). The accession IDs for Experiment 1 can be found in Figure 2A. The accession IDs for Experiment 2 are SRR13130411 and SRR12344432 that represent *K. pneumoniae* and healthy gut metagenome, respectively.

The scripts for generating the artificial metagenomes with the various tools used in this study can be found in the supplementary materials and on FigShare:

Supplementary materials S1 – https://doi.org/10.6084/m9.figshare.14774781

Supplementary materials S2 – https://doi.org/10.6084/m9.figshare.14774784

Data generated by the MG-RAST pipeline used in Experiment 3 is publicly available through their online repository at:

https://www.mg-rast.org/mgmain.html?mgpage=project&project=mgp102827

## Supplementary Material

lqac050_Supplemental_Files
